# Plant-Derived Antioxidants as Modulators of Redox Signaling and Epigenetic Reprogramming in Cancer

**DOI:** 10.3390/cells14241948

**Published:** 2025-12-08

**Authors:** Thi Thuy Truong, Alka Ashok Singh, Soonhyuk Tak, Sungsoo Na, Jaeyeop Choi, Junghwan Oh, Sudip Mondal

**Affiliations:** 1 Industry 4.0 Convergence Bionics Engineering, Department of Biomedical Engineering, Pukyong National University, Busan 48513, Republic of Korea; thuytt2405@gmail.com (T.T.T.); soontak@pukyong.ac.kr (S.T.); 2 Department of Life Sciences, Yeungnam University, Gyeongsan 38541, Republic of Korea; alkasingh10f@yu.ac.kr; 3 Weldon School of Biomedical Engineering, Purdue University, Indianapolis, IN 46202, USA; na19@purdue.edu; 4 Smart Gym-Based Translational Research Center for Active Senior’s Healthcare, Pukyong National University, Busan 48513, Republic of Korea; jaeyeopchoi@pknu.ac.kr; 5 Digital Healthcare Research Center, Institute of Information Technology and Convergence, Pukyong National University, Busan 48513, Republic of Korea

**Keywords:** plant-derived antioxidants, redox signaling, epigenetic reprogramming, cancer therapy, ROS modulation

## Abstract

Redox imbalance and epigenetic dysregulation, which both contribute to tumor initiation, survival, and resistance to therapy, are intimately linked to the progression of cancer. Reactive oxygen species (ROS) have two contrasting effects: at moderate concentrations, they promote angiogenesis and oncogenic signaling, whereas at high concentrations, they trigger apoptosis. Oxidative stress alters histone modifications, DNA methylation, and non-coding RNA (ncRNA) expression, reshaping the epigenetic landscape and supporting malignant phenotypes. Plant-derived antioxidants, including flavonoids, polyphenols, alkaloids, and terpenoids, act as dual modulators of cancer biology. They scavenge or regulate reactive oxygen species (ROS), restore redox balance, activate tumor suppressor pathways, inhibit oncogenic mechanisms, and reverse abnormal epigenetic marks. Compounds such as resveratrol, curcumin, epigallocatechin gallate (EGCG), quercetin, and sulforaphane modulate DNA methyltransferases (DNMTs), histone deacetylases (HDACs), and non-coding RNA networks, and can enhance chemotherapy and radiation therapy. Despite promising mechanisms, challenges remain in translational efficacy, optimal dosing, and bioavailability. This review emphasizes the potential of plant-derived antioxidants as precision oncology adjuncts and highlights the need for biomarker-guided strategies, nano-delivery systems, and clinical validation to fully realize their therapeutic benefits. Plant-derived antioxidants mitigate ROS-induced oncogenic signaling, as evidenced by in vitro and clinical models.

## 1. Introduction

Cancer persists to stand as one of the leading causes of morbidity and mortality worldwide. Current treatment approaches, such as immunotherapy, radiotherapy, chemotherapy, and surgery, are frequently constrained by toxicity, resistance, and recurrence. It is anticipated that this trend will continue, which is evidence of the advancements in the prevention, diagnosis, and treatment of the disease over the past 50 years. It would be a mistake to let optimism about our accomplishments in this and other highly developed nations overshadow the reality of the global cancer problem [1]. One of the fundamental hallmarks of cancer is redox imbalance, in which reactive oxygen species (ROS) play two distinct roles in tumor biology. Interestingly, ROS has a dynamic influence on the tumor microenvironment and has been shown to initiate cancer angiogenesis, metastasis, and survival at varying concentrations. At moderate concentrations, ROS activates the cancer cell survival signaling cascade involving mitogen-activated protein kinase/extracellular signal-regulated protein kinases 1/2 (MAPK/ERK1/2) [2], p38, c-Jun N-terminal kinase (JNK), and phosphoinositide-3-kinase/protein kinase B (PI3K/Akt), which in turn activate the nuclear factor kappa-light-chain-enhancer of activated B cells (NF-κB) [3], matrix metalloproteinases (MMPs), and vascular endothelial growth factor (VEGF). ROS, at high concentrations, can induce cancer cell apoptosis [4]. As a result, ROS levels have a significant impact on whether tumorigenesis is enhanced or apoptosis occurs [5]. This “ROS paradox” highlights the importance of redox regulation in cancer research [6]. The study of heritable modifications in gene expression that take place apart from alterations in the primary DNA sequence is known as epigenetics. According to recent studies, epigenetic abnormalities are just as important in the development of cancer as genetic changes [7]. The effects of non-coding RNA, histone tail modifications, and DNA methylation are examples of epigenetic changes, even though the DNA sequence remains unchanged. By changing the genome’s compactness and, consequently, accessibility to various regulatory proteins, these alterations are necessary to control the cell’s normal functioning [8]. The role and effectiveness of first-line defense antioxidants, which primarily include superoxide dismutase (SOD), catalase (CAT), and glutathione peroxidase (GPX), is critical and indispensable in the overall antioxidant defense strategy, particularly in relation to the superoxide anion radical (*O_2_), which is constantly generated in normal body metabolism, particularly through the mitochondrial energy production pathway (MEPP) [9]. Excess ROS in oxidative stress conditions can damage cellular proteins, lipids, and DNA, resulting in cell damage that may contribute to carcinogenesis. Several studies have demonstrated that cancer cells respond to oxidative stress by upregulating the expression of antioxidant enzymes and molecules. In cancer biology, reactive oxygen species (ROS) play a double-edged regulatory function. ROS can activate pro-survival pathways like PI3K/Akt and NF-κB, leading to cell proliferation and angiogenesis [10,11]. However, excessive ROS generation outperforms cellular antioxidant defense, resulting in oxidative modification of DNA, proteins, and lipids, which causes genomic instability and apoptosis. This redox imbalance is caused by mitochondrial dysfunction, NADPH oxidase activity, and metabolic reprogramming induced by oncogenes. Importantly, prolonged oxidative stress can change the epigenetic landscape by altering DNA methylation and histone acetylation patterns, linking redox imbalance to transcriptional reprogramming during tumor progression [12]. This poses a problem for treatment because, although antioxidants can prevent the growth of tumors, they may also shield cancer cells from the oxidative damage brought on by conventional treatments.

**Figure 1:**
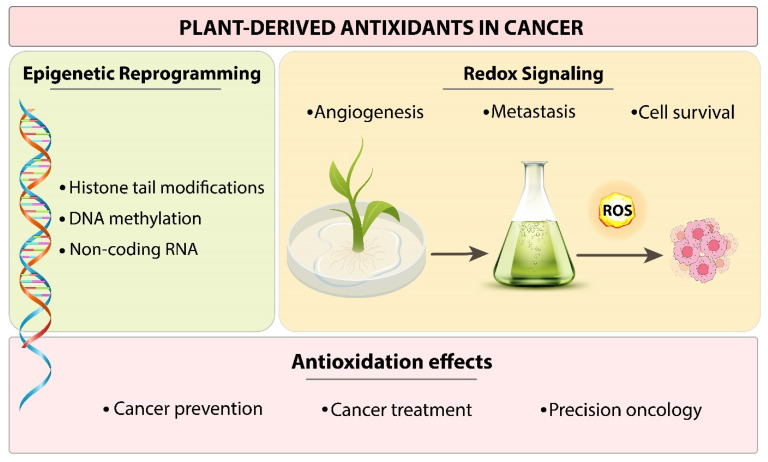
Plant-derived antioxidants play dual roles in cancer by modulating epigenetic and redox pathways, as illustrated schematically.

In recent years, plant-derived antioxidants such as polyphenols, flavonoids, alkaloids, and terpenoids have received attention for their potential as cancer prevention and treatment agents [13]. Phytochemicals have been shown to directly scavenge ROS and increase the expression of cellular antioxidant enzymes, thus protecting against oxidative stress-induced cellular injury [14]. Carcinogen-induced reactive metabolites and oxidative stress can cause genetic mutations, genomic instability, neoplastic transformation, and, eventually, carcinogenesis. In both preclinical animal models and human epidemiological studies, numerous dietary phytochemicals found in vegetables and fruits have been shown to have cancer-chemo preventive effects. These phytochemicals may inhibit carcinogenesis by either directly scavenging reactive oxygen species/reactive nitrogen species (ROS/RNS) inducing cellular defense detoxifying/antioxidant enzymes [15]. In preclinical and clinical studies, natural compounds like curcumin, resveratrol, epigallocatechin gallate (EGCG), quercetin, and sulforaphane have shown promise in reducing treatment-associated toxicity, inhibiting tumor growth, and improving the effectiveness of chemotherapy [16]. This review will primarily focus on the dual function of antioxidants derived from plants in regulating both epigenetic reprogramming and redox signaling in cancer (Figure 1).

## 2. Redox Biology in Cancer

**Figure 2:**
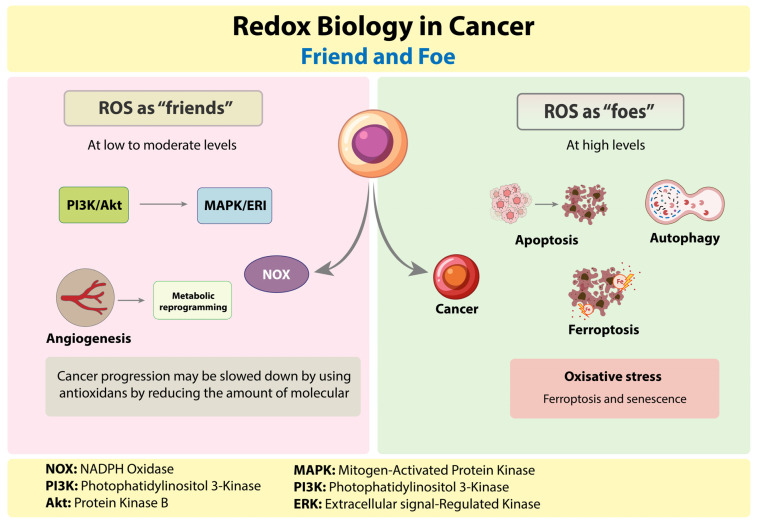
ROS plays a dual role in cancer. ROS, at low to moderate levels, promote survival, angiogenesis, and metabolic reprogramming via PI3K/Akt and MAPK/ERK signaling. ROS at high concentrations cause oxidative stress, which leads to apoptosis, autophagy, and ferroptosis.

According to mouse functional genetics and patient genomics, senescence prevents prostate cancer from spreading to other areas [17]. While many efforts concentrate on eradicating senescent cells, others seek to identify unique traits that differentiate them from aging and normal cells. Using the knowledge of the redox sensitivity of proliferating cancer cells as an analogy, study described how investigation of the redox state of senescent cells may aid in the definition of novel markers and pro-oxidant vulnerabilities [18]. During respiration and photosynthesis, mitochondria, membrane-bound NADPH oxidases (NOXs), peroxisomes, and chloroplasts are the main locations where ROS are produced. During the electron transfer reactions in the mitochondrial electron transport chain, oxidative phosphorylation generates ATP. An imbalance in ROS leads to a number of pathological conditions, including cancer, which is linked to elevated ROS levels that promote the growth and spread of tumors. Cancer cells effectively regulate the antioxidative pathways to prevent the excessive oxidative damage caused by ROS, thereby favoring their own survival and maintenance. Research on the significance of ROS has been ongoing in “cancer stem cells” (CSCs), a subset of cancer cells that possess characteristics and attributes similar to those of stem cells [19]. Reactive oxygen species cause genetic instability by damaging DNA or increasing the number of mutations. ROS exposure affects transcription factors like Sp1, AP1, and NF-κβ, which play roles in cancer stem cell maintenance, metastasis, and proliferation. ROSs may play a role in a variety of cancer-related processes, including apoptosis, angiogenesis, metastasis, and inflammation (Figure 2) [20]. Enzymatic and non-enzymatic molecules make up the body’s complex antioxidant defense system, which fights off free radicals and safeguards important biomolecules.

Through pathways like HIF-1α, YAP1, and NF-κB, ROS in the tumor microenvironment can control PD-L1 expression on cancer cells, frequently encouraging its upregulation. While ROS scavenging typically lowers PD-L1 levels, elevated PD-L1 may aid in immune evasion by inhibiting cytotoxic T-cell activity. However, depending on particular modulators and cellular targets, the impact of ROS on PD-L1 can change depending on the context [21].

### 2.1. ROS Activity on Cancer

At low-to-moderate levels, ROS activate survival pathways like PI3K/Akt, MAPK/ERK, HIF-1α, and NF-κB [22]. These signals promote cell proliferation, angiogenesis, metabolic reprogramming, and epithelial–mesenchymal transition (EMT), all of which are hallmarks of cancer [23]. ROS may be produced by tumor cells in excess of their antioxidant or DNA repair capacity, and these endogenous ROS may be able to damage DNA and cause mutations in important genes involved in DNA stability. It is intriguing that oxidative DNA damage might be a major contributor to the genomic instability seen in human cancers, as antioxidant therapy may be able to reduce the extent of oxidative damage. Cancer progression may be slowed down by using antioxidants by reducing the amount of molecular damage caused by ROS [24].

### 2.2. ROS Activity as Anticancer

At high levels, ROS overwhelm the cellular antioxidant defenses and induce oxidative stress [25]. This causes severe DNA damage, lipid peroxidation, protein misfolding, and mitochondrial dysfunction [26]. Such stress activates cell death pathways such as apoptosis, autophagy, and ferroptosis, which can inhibit tumor growth [27]. By taking advantage of redox processes, neutrophils have become important players in the development of tumors. They emit reactive oxygen species (ROS), which damage DNA and promote carcinogenesis and genetic instability. Neutrophils release pro-inflammatory molecules like miR-23a and miR-155, as well as neutrophil elastase (NE) and prostaglandin E2 (PGE2), all of which work together to stimulate the growth of tumor cells. Neutrophils prevent tumor cells from senescence by generating interleukin-1 receptor antagonist (IL-1RA), and the release of NE, TGF-β, and IL-17 promotes the epithelial–mesenchymal transition (EMT), which in turn promotes angiogenesis and metastasis. Therefore, ROS produced by neutrophils act as potent “enemies”, promoting the development and spread of tumors [28].

To survive in this paradoxical environment, tumor cells hijack and rewire the endogenous antioxidant systems:

The antioxidant enzymes known as superoxide dismutases (SOD) were first thought to be responsible only for detoxifying superoxide radicals. Subsequent research showed that mitochondrial SOD (MnSOD) controls basic cellular processes in addition to oxidative damage, and that low MnSOD activity is associated with faster growth of cancer cells. Since hydrogen peroxide and superoxide are now acknowledged as crucial signaling molecules, MnSOD is positioned as a key regulator in cellular redox biology [29]. Hydrogen peroxide can be detoxified by glutathione peroxidase (GPx) and catalase [30]. On the other hand, thioredoxin and peroxiredoxins keep redox-sensitive proteins in their reduced state [31]. Due to biomolecule deregulation, a high level of ROS promotes carcinogenesis in cells with defective signaling factors. In this line, NRF2 appears to act as a master regulator, protecting cells from oxidative and electrophilic stress. Nrf2 is an intracellular transcription factor that controls the expression of several genes that produce anti-oxidative enzymes, detoxifying factors, anti-apoptotic proteins, and drug transporters [32].

### 2.3. This Dynamic Creates the “Redox Paradox” of Cancer

ROS are essential for cancer initiation and progression. Excessive ROS are lethal, indicating a vulnerability that therapies can exploit [4,33]. Normal cells have oxygen levels ranging from 3.1% to 8.7%; however, tumor cells can have levels as low as 0.01%, making hypoxia a common feature of many solid tumors. In low-oxygen conditions, hypoxia-inducible factor-1 (HIF-1) activates and promotes the transcription of angiogenic factors (e.g., VEGF, PDGF, TGF-α) by recognizing a consensus hypoxia response element in their promoter regions, which is necessary for tumor angiogenesis [34]. HIF-1-mediated transcriptional control of angiogenic factors is regulated by alterations in the redox state of tumor cells and the surrounding microenvironment (for example, when reactive oxygen species, or ROS, are produced). These alterations also affect downstream angiogenesis signaling, which is where receptor-competent isoforms of angiogenic factors influence tumor angiogenesis. An ideal design of angiogenesis-targeted therapies thus requires an understanding of the entire range of redox regulations of tumor angiogenesis signaling and their mediators [35]. As a result, redox signaling targeting requires careful balancing. Therapeutic approaches must carefully control oxidative stress, either by stimulating ROS to drive cancer cells into death pathways or by employing antioxidants to reduce inflammation and tumor initiation. Because of this, redox biology is a crucial and contradictory node in cancer treatment.

## 3. Epigenetic Reprogramming in Cancer

**Figure 3:**
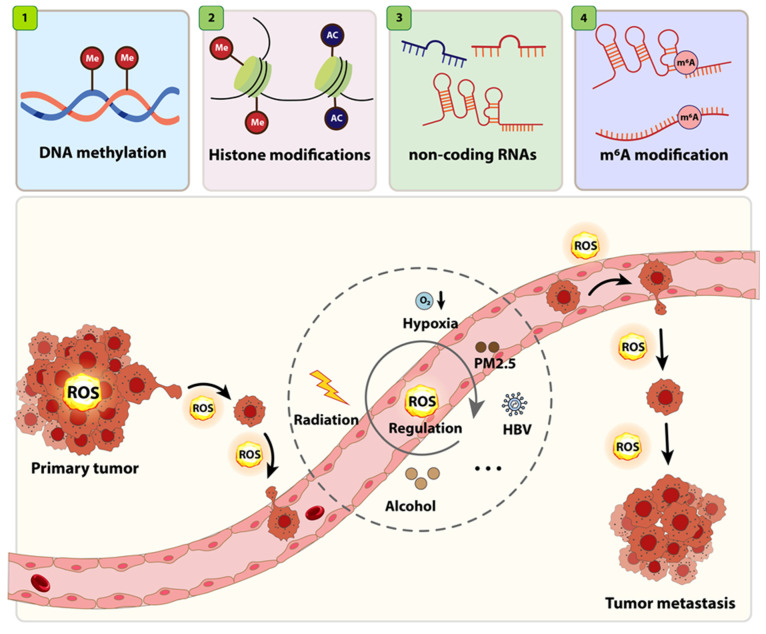
ROS-induced epigenetic reprogramming in cancer. Cancer progression is influenced by both genetic mutations and epigenetic dysregulation. The top panels show key epigenetic mechanisms, including DNA methylation, histone modifications, non-coding RNAs (miRNAs and lncRNAs), and m6A RNA modifications. Modified from Peng et al. [38]. Licensed under Creative Commons Attribution (CC BY 4.0).

Cancer is caused by both genetic mutations and epigenetic dysregulation, whereby reversible alterations in gene expression lead to abnormal transcription, initiation, progression, drug resistance, and metastasis (Figure 3). As a result, epigenetic mechanisms are promising targets for therapeutic intervention [36]. Significantly, oxidative stress (ROS) is a major cause of these epigenetic changes, establishing a connection between redox imbalance and the development of cancer [37].

### 3.1. DNA Methylation: Silencing of Tumor Suppressors

DNA methyltransferases (DNMT1, DNMT3A, and DNMT3B) mediate DNA methylation, which mostly takes place at CpG islands within gene promoters [39]. Tumor suppressor genes (such as p16, BRCA1, PTEN, and MLH1) are silenced in cancer by hypermethylation, whereas oncogene activation and genomic instability are caused by hypomethylation [40]. ROS-induced oxidative stress is linked to both abnormal hypermethylation of tumor suppressor gene (TSG) promoter regions and overall hypomethylation. The oxidized DNA lesion 8-hydroxy-2′-deoxyguanosine (8-OHdG) can cause DNA hypomethylation by inhibiting DNA methylation at nearby cytosine bases, whereas another oxidized DNA lesion, 5-hydroxymethylcytosine (5hmC), can activate DNA demethylation processes, resulting in DNA hypomethylation [41].

ROS signaling has the greatest effect on DNA methylation in cancer [41]. Certain tumor suppressor genes have been demonstrated to be silenced by ROS-dependent DNA methylation, which also starts the subsequent growth of tumors. For instance, in hepatocellular carcinoma, extended exposure to ROS caused methylation of CpG island II on the cadherin promoter. Age-related DNA hypomethylation has many examples. For instance, it has been demonstrated that aging alters the genome’s distribution of 5-methylcytosine, a product of DNA methylation, which lowers global DNA methylation [42]. Targeting these events could result in the creation of innovative therapeutic approaches for the prevention of human skin cancers because epigenetic changes are reversible. For instance, hypermethylation and silencing of tumor suppressor promoters are associated with elevated ROS levels in glioblastoma [43].

### 3.2. Histone Modifications: Acetylation/Methylation Imbalance

The various post-translational changes that histones go through—acetylation, methylation, phosphorylation, and ubiquitination—control the structure of the chromatin and the expression of genes [44]. Histone acetyltransferases (HATs) catalyze histone acetylation, which reduces the strength of histone–DNA interactions by neutralizing the positive charge of lysine residues. This promotes active gene transcription by establishing a relaxed or open chromatin structure that makes nucleosomal DNA accessible to transcription factors and coactivators. On the other hand, transcription is suppressed and chromatin compaction is restored when histone deacetylases (HDACs) remove these acetyl groups [45]. HDAC overexpression promotes proliferation and inhibits tumor suppressor genes in cancer [46]. ROS affect histone-modifying enzymes, such as histone methyltransferases (HMTs) and HDACs, which are activated by oxidative stress [47]. This results in the activation of survival pathways and the transcriptional repression of genes linked to apoptosis [48]. For instance, EMT and metastasis have been linked to ROS-driven histone H3K9 methylation [38].

### 3.3. Non-Coding RNAs (miRNAs, lncRNAs) in Redox Regulation

Non-coding RNAs (ncRNAs), such as long non-coding RNAs (lncRNAs) and microRNAs (miRNAs), have become important modulators of cancer biology and redox homeostasis [49,50]. These RNAs function as molecular switches that detect oxidative stress and regulate networks of gene expression related to metabolism, apoptosis, and cell division [51]. Significantly, reactive oxygen species (ROS) and ncRNAs regulate each other in both directions. While ncRNAs modulate ROS-generating and ROS-scavenging pathways, ROS alters the transcription and stability of ncRNAs, creating feedback loops that strengthen tumorigenic signaling [52,53]. Given its capacity to inhibit the functions of multiple tumor suppressor genes and to stimulate the growth, invasion, and metastasis of tumor cells, microRNA-21 (miR-21) is regarded as an onco-microRNA. It has recently been discovered that transforming growth factor-beta (TGF-β) up-regulates miR-21 expression, and elevated miR-21 expression is commonly observed in breast cancer [54]. In colorectal cancer, miR-34a suppresses tumor growth. It causes G1 cell cycle arrest in a p53-dependent manner, induces apoptosis, and suppresses cell migration, invasion, and growth [55]. Hox transcript antisense intergenic RNA (HOTAIR), a long non-coding RNA, is predictive of a poor prognosis in colorectal and pancreatic cancers and has recently been linked to breast cancer metastasis. Despite the fact that polycomb repressive complex 2 (PRC2) and lysine specific demethylase 1 (LSD1) have been shown to be functional targets of HOTAIR, it is still unclear how HOTAIR controls the progression of the glioma cell cycle. According to the study, HOTAIR’s 5′ domain binds to the PRC2 complex, promoting glioma cell cycle progression [56]. MALAT1 is a long non-coding RNA that is significantly upregulated in non-small cell lung cancer (NSCLC) cell lines such as A549, H23, H522, H1299, and H460 when compared to normal bronchial epithelial cells. MALAT1 knockdown increases miR-124 levels, whereas miR-124 mimics suppress MALAT1, indicating a negative correlation between the two. Functionally, MALAT1 promotes NSCLC cell proliferation and colony formation, which miR-124 can reverse. MALAT1 could act as a competing endogenous RNA (ceRNA) to regulate the miR-124/STAT3 axis. MALAT1 modulates miR-124 and STAT3, which contribute to the development of NSCLC [57].

#### 3.3.1. MicroRNAs (miRNAs)

MicroRNAs (miRNAs) are essential regulators of immunological response and tumor development. Since the immune system plays a crucial role in identifying and eliminating cancerous cells, it is now crucial to comprehend how miRNAs affect immune responses in order to advance cancer treatment [58]. miRNAs are short non-coding RNAs (~22 nucleotides) that bind to complementary sequences in target mRNAs’ 3′ untranslated regions (UTRs) to cause translational repression or degradation [59,60]. In tumor cells, oxidative stress dramatically alters the profiles of miRNA expression [61]. The majority of human tumors overexpress microRNA-21 (miR-21), an oncomir that acts on several targets to encourage the growth and spread of cancer. The role of miR-21 in cancer by demonstrating how it controls the production of ROS, which encourage the growth of new tumors. TNFα and SOD3 were important targets of miR-21 in mediating this function [62]. MiR-21 confers resistance to apoptosis and promotes tumor survival by suppressing pro-apoptotic targets like PTEN and PDCD4 [63]. On the other hand, when ROS levels are high, tumor-suppressive miRNAs like miR-34a, which typically target oncogenes like BCL2 and SIRT1 to cause cell cycle arrest and apoptosis, are frequently downregulated [64]. This imbalance causes the redox-sensitive regulatory network to support tumor growth and resistance to chemotherapeutics. The epithelial–mesenchymal transition (EMT), angiogenesis, and metabolic reprogramming in cancer cells have all been connected to the oxidative stress-induced modification of miRNAs like the miR-200 family and miR-210 [65].

#### 3.3.2. Long Non-Coding RNAs (lncRNAs)

Long non-coding RNAs (lncRNAs) are transcripts that regulate gene expression through a variety of mechanisms, including chromatin remodeling, transcriptional interference, and protein stability modulation [66]. Numerous long non-coding RNAs (lncRNAs) play a role in oncogenesis and are dynamically regulated by redox condition [67]. For example, oxidative stress causes the upregulation of HOTAIR, a lncRNA linked to chromatin reprogramming, which enhances EMT and changes histone methylation patterns to promote metastasis [68]. LncRNA H19 prevents cancer cells from experiencing oxidative stress. VEGF signaling is activated and glioma angiogenesis follows when H19, a ceRNA that inhibits miR-138, is upregulated in glioma cells [69]. Inhibiting lncRNA MALAT1 has been shown to increase cellular ROS levels. HCC cells upregulated MALAT1 to promote VEGF-A expression and angiogenesis through sponging miR-140 [70]. A large number of redox-sensitive lncRNAs integrate oxidative signals into the cancer transcriptome by directly interacting with transcription factors like NRF2, mitochondrial regulators, or genes involved in antioxidant defense [71,72].

#### 3.3.3. Feedback Loops Between ROS and ncRNAs

ROS not only regulate ncRNA expression, but also influence it in a reciprocal manner. For example, ncRNAs that suppress antioxidant genes (such as SOD2, catalase, or glutathione peroxidases) cause long-term ROS accumulation, which promotes ncRNA dysregulation [73,74]. These self-reinforcing circuits initiate a vicious cycle in which ROS and ncRNAs work together to promote malignant transformation, therapy resistance, and metastasis [75]. These redox–ncRNA feedback loops present fresh chances for therapeutic action [76]. Redox-sensitive miRNA mimics, lncRNA inhibitors, and ncRNA-targeting nanoparticles are examples of therapies that aim to restore redox balance in tumors [77].

### 3.4. ROS as Drivers of Epigenetic Alterations

Reactive oxygen species have a significant impact on the epigenetic landscape [78]. ROS have a significant impact on gene expression patterns without changing the underlying DNA sequence by causing direct chemical modifications of DNA and histones or indirectly regulating epigenetic enzymes. These epigenetic changes not only promote tumor initiation and progression, but also maintain redox imbalance, resulting in a self-sustaining oncogenic environment [78].

#### 3.4.1. Direct Epigenetic Modifications by ROS

One of the most studied oxidative DNA lesions is 8-oxo-2′-deoxyguanosine (8-oxo-dG), which occurs when guanine bases are oxidatively modified [79]. This lesion disrupts DNA methylation patterns and causes aberrant gene silencing or activation by changing the binding affinity of methyl-CpG-binding proteins (e.g., MeCP2) [80]. Histone carbonylation, a non-enzymatic alteration that modifies histone charge and structural flexibility, is also brought on by oxidative stress [81,82]. These modifications impair chromatin compaction, which can lead to tumor suppressors being repressed or oncogenes exhibiting aberrant transcriptional activity [82]. ROS-induced breaks in DNA strands attract DNA repair machinery, which frequently interacts with chromatin modifiers to modify local epigenetic states [83].

#### 3.4.2. Indirect Regulation via Epigenetic Enzymes

ROS function as mediators of signaling that alter the activity of important epigenetic enzymes [84]. For example, oxidative stress increases the activity of DNA methyltransferases (DNMTs), which causes transcriptional silencing and hypermethylation of the promoters of tumor suppressor genes [85]. Similarly, ROS promote a transcriptionally repressive chromatin environment that supports oncogenesis by activating histone methyltransferases (HMTs) and histone deacetylases (HDACs) [86,87,88]. On the other hand, TET enzymes, which catalyze the transformation of 5-methylcytosine (5-mC) into 5-hydroxymethylcytosine (5-hmC), are compromised by ROS [89]. A common feature of many cancers is global hypohydroxymethylation, which is caused by decreased TET activity [90]. Together, these alterations rewire the epigenome, promoting uncontrolled growth, stemness, and dedifferentiation.

#### 3.4.3. ROS-Epigenetics Feedback in the Development of Tumors

Oxidative stress is a state in which the production of reactive oxygen species (ROS) surpasses the cell’s capacity to metabolize them, leading to an excessive build-up of ROS that overwhelms the cell’s defenses. Such a state has been demonstrated to control the genetic and epigenetic cascades that underlie the changed expression of genes in human diseases, such as cancer [91]. ROS and epigenetic regulation interact in a two-way fashion. ROS accumulation is maintained by the epigenetic silencing of antioxidant genes (e.g., SOD, GPX, and catalase) through promoter hypermethylation, and metabolic and oxidative stress are further intensified by ROS-driven activation of oncogenic transcriptional programs [38]. Thus, a vicious cycle is created: By altering metabolism and causing mitochondrial dysfunction, ROS cause epigenetic reprogramming, which promotes tumor growth and metastasis. Tumor growth also produces more ROS [4,38]. Reversing ROS-induced epigenetic marks or focusing on redox-sensitive epigenetic enzymes are two new oncology treatment approaches that aim to break this cycle [71].

## 4. Plant-Derived Antioxidants as Redox Modulators in Cancer

### 4.1. Polyphenols

Polyphenols are a diverse group of plant-derived compounds with multiple phenolic groups [92]. They have been extensively studied for their antioxidant, anti-inflammatory, and anticancer properties [93]. They have an impact on ROS levels, signaling pathways, and epigenetic regulation in cancer by acting as redox modulators [94]. According to recent data, polyphenols’ function in regulating redox homeostasis (i.e., pro/antioxidative effect) in cancer cells is connected to this antitumor activity. A disruption in redox homeostasis may result in an excess of reactive oxygen species (ROS), which causes oxidative stress. Oxidative stress is crucial for many aspects of tumors, including drug resistance, tumorigenesis, and progression [95]. In cancer cells, moderate ROS (ROS levels that promote tissue turnover and cell proliferation) act as second messengers in cellular physiological processes, modulating cellular signaling and biological reactions to maintain endogenous homeostasis [96]. Thus, offering benefits for cell survival, metastasis, and carcinogenesis. Nevertheless, too many ROS above the toxic threshold-the highest amount appropriate for maintaining cellular homeostasis, which sets off redox homeostasis to initiate cell death-may hinder the growth of tumors and cause cell senescence, apoptosis, or ferroptosis [97,98]. Thus, using free radicals or antioxidants to control cellular redox homeostasis is important for cancer treatment. Preclinical studies and clinical evaluations have extensively examined the use of natural antioxidants and pro-oxidants to modulate redox homeostasis for the treatment of cancer in recent years [66,67,68,69], and numerous clinical trials have been conducted (NCT01912820, NCT03493997, NCT00256334, NCT00433576, NCT01717066). Being the primary natural antioxidants, polyphenols have garnered a lot of attention as new anticancer drugs that can also control oxidative stress based on their characteristics and dosage in various tumor models [99,100]. The potential of plant-derived antioxidants to reduce oxidative stress, increase patient tolerance, and affect cancer-related biomarkers has been the subject of numerous clinical studies. A summary of these trials is shown in Table 1.

Polyphenols like resveratrol (phytoalexin, a flavonoid in red wine) and curcumin (diferuloylmethane, an active ingredient in turmeric) can directly scavenge ROS, alter signaling pathways mediated by MAP kinase and NF-κB, and activate the Nrf2 gene to upregulate glutathione biosynthesis [101]. Through the inhibition of histone acetyltransferase activity and the activation of histone deacetylase/sirtuins, they also suppress the expression of pro-inflammatory mediators, matrix metalloproteinases, adhesion molecules, and growth factor receptor genes. Therefore, these polyphenolic compounds are useful as anti-inflammatory and antioxidant treatments for chronic inflammatory diseases that are epigenetically regulated [102]. Through the prolonged activation and phosphorylation of MAP kinases and redox-sensitive transcription factors, including AP-1 and NF-κB, reactive oxygen species contribute significantly to the escalation of inflammation in a variety of inflammatory diseases [103].

**Figure 4:**
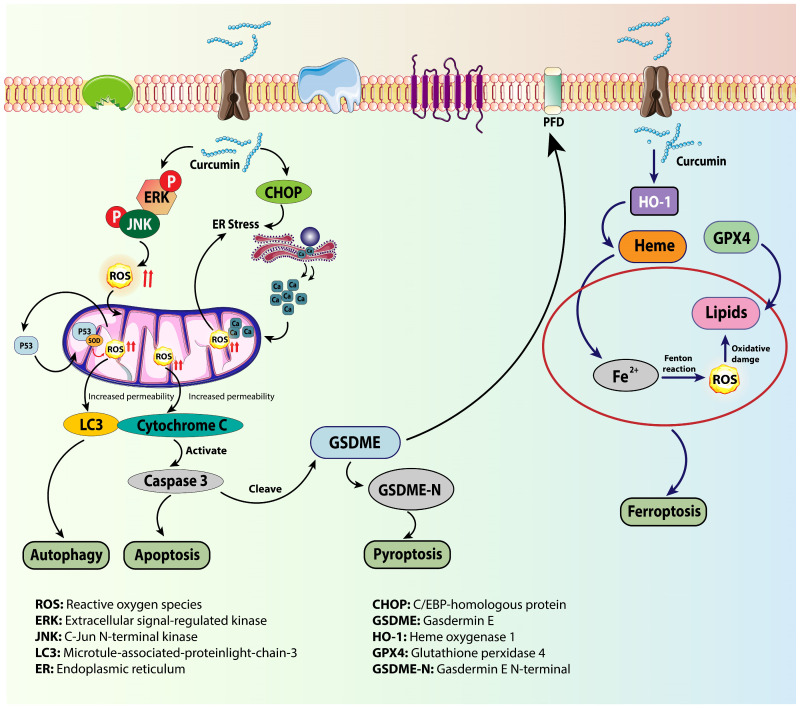
Diagrammatic representation of curcumin regulates the pathways leading to cell death. Curcumin causes ER stress and activates JNK/ERK signaling, which leads to the buildup of ROS and mitochondrial dysfunction. Cytochrome C and caspase-3 then trigger autophagy and apoptosis. It alters HO-1/GPX4 signaling to affect ferroptosis and lipid peroxidation, whereas pyroptosis is caused by caspase-3-mediated GSDME cleavage. These interrelated processes demonstrate curcumin’s complex regulation of oxidative stress-induced cell fate. Reproduced from Hu et al. [117]. Licensed under Creative Commons Attribution (CC BY 4.0).

Curcumin, also known as diferuloylmethane, is a yellow-colored polyphenolic pigment that is derived from the rhizome of Curcuma longa Linn (Family-Zingiberaceae) and belongs to the curcuminoid family of compounds. It is the main and active ingredient in turmeric. It has been reported that curcumin’s hydroxyl and methoxy groups provide anti-carcinogenic and antioxidant properties, respectively (Figure 4). Although the intestinal mucosa and liver metabolize curcumin, the gastrointestinal tract does not change about 40–85% of the total amount consumed. Ten Humans have been shown to be completely safe when consuming up to 10 g of curcumin per day [104]. It has been discovered that when taken with piperin, an active component of black pepper, its bioavailability is increased 20-fold [105]. In a mouse model of acute kidney injury caused by sepsis, tetrahydrocurcumin improved kidney function and reduced renal histological damage, decreased inflammatory response (IL-1β, IL-6, and TNF-α), reduced oxidative stress (as indicated by MDA level, SOD, GSH, CAT, and GPx activities), and prevented cell apoptosis in septic mice’s renal tissues by increasing SIRT1 expression and decreasing downstream molecules Ac-p65 and Ac-foxo1 [106]. Curcumin decreased tubular cell apoptosis, decreased oxidative stress, and increased SIRT1 and Nrf2/HO-1 expression in a rat model of acute kidney injury caused by gentamicin [107]. In mouse and cell models of iron overload, curcumin reduced iron loading-induced autophagy and increased SIRT3 expression, which is linked to a decrease in SOD activity and protection against oxidative stress [108]. All these effects of curcumin could be extrapolated to different pathologies including cancer. Table 2 summarizes the sources, mechanisms, and effects of several plant-derived antioxidants that have been demonstrated to alter redox signaling and epigenetic mechanisms, thereby impacting cancer-related outcomes.

**Table 2:** Antioxidants derived from plants, their redox targets, epigenetic effects, and cancer outcomes.

Compound	Source (Plant/Food)	Redox Modulation Mechanism	Epigenetic Regulation	Cancer-Related Outcomes	References
Curcumin	Turmeric (Curcuma longa)	Anti-inflammatory and antioxidant properties.	Reduced expression of DNMT1; hypomethylation of the RASSF1 promoter; hypomethylation of the RARβ promoter; and hypomethylation of the Nrf2 promoter.	Tumor suppressor gene reactivation, reduced tumor size in mammary carcinoma, and prevention of lung cancer progression Acute myeloid leukemia impact; decreased development of prostate cancer.	[109]
Genistein	Soy and other plant-based legumes	Anti-inflammatory, antioxidant, and prognostic properties.	Reduced DNMT1 expression results in ERα reactivation; downregulation of DNMT3 results in CDH5 promoter hypomethylation.	Reduced tumor growth in neuroblastoma; inhibition of DNA methyltransferase activity; and reactivation of the estrogen receptor in breast cancer.	[109,110]
Quercetin	Apple, leafy vegetables, and onions	Antioxidant in healthy cells, pro-oxidant in cancer cells.	regulates histone acetylation and modifies PDCD4 and miR-21.	prevents invasion and metastasis and triggers apoptosis.	[111]
EGCG Epigallocatechin-3-gallate (green tea)	Green tea	eliminates ROS and controls the Nrf2/KEAP1 pathway.	Histone acetylation, DNMT inhibition, and TSG reactivation.	Reversal of drug resistance, apoptosis, and cell cycle arrest.	[112]
Resveratrol	Grapes, red wine	Inhibits pro-inflammatory cytokines (TNF-α, IL-17); affects fatty acid oxidation, mitochondrial biogenesis, and gluconeogenesis; suppresses NF-κB activity; inhibits cytochrome P450 and cyclooxygenase; and modulates ROS.	Histone acetylation, DNMT inhibition, and ncRNA modulation.	Potential anticancer effects include inhibiting the NF-κB pathway linked to cancer, suppressing pro-inflammatory cytokines, and inducing apoptosis in activated T cells; these measures may stop tumor growth and inflammation-induced carcinogenesis.	[113,114]
Berberine	Traditional Chinese herbal remedies, such as those made from Berberis species	reduces oxidative stress in healthy cells and produces ROS in tumor cells.	inhibits topoisomerase and telomerase and can bind to oligonucleotides to stabilize DNA triplexes or G-quadruplexes.	inhibits the growth, carcinogenesis, and metastasis of tumors; inhibits the growth of several tumor types by blocking cancer pathways such as NF-κB and MAPK.	[113]
Sulforaphane,	Broccoli, cruciferous vegetables	Dual redox function: causes ROS in cancer cells and activates Nrf2 in healthy cells.	DNA methylation modulation, miRNA regulation, and HDAC inhibition.	Reactivation of tumor suppressors and induction of apoptosis.	[115,116]

#### EGCG Epigallocatechin-3-Gallate (Green Tea)

Polyphenols are the primary bioactive molecules in tea. Polyphenols can be found in dried tea extract at a concentration of 25–40% [118]. Tea’s primary polyphenolic compounds are flavan-3-ols known as catechins. Catechins include epigallocatechin-3-gallate (EGCG), epicatechin-3-gallate, and epicatechin, as well as gallocatechins and gallocatechin gallate. EGCG is the most common [8]. Epigallocatechin-3-gallate (EGCG), one of the main ingredients in green tea, has the strongest anticancer properties. The majority of research shows that EGCG’s suppressive mechanisms modify the cancer cell cycle, development, and apoptosis by activating or inhibiting multiple signal pathways. The epigenetic alteration caused by DNA methylation or methyltransferases, histone acetylation or deacetylases, and no coding RNAs (micoRNAs) is another mechanism that explains the various effects of EGCG in cancer [119]. The study found that ROS plays a role in cancer development by activating various inflammatory pathways such as NF-KB and the Nrf2-KEAP1 pathway, as well as making cancer cells resistant to immune cells. Polyphenols’ ability to modulate ROS as antioxidants, allowing for the synthesis of Phase II detoxification and antioxidant enzymes, or as pro-oxidants, increasing ROS levels and sensitizing cancer cells to drugs, has been documented [112]. It has been demonstrated that EGCG stimulates the expression of several enzymes, including hemeoxygenase-1, glutathione S-transferase, glutathione peroxidase, and glutamate cysteine ligase. These enzymes play a role in the removal or deactivation of electrophiles and reactive oxygen species linked to multi-stage carcinogenesis. Nuclear factor erythroid 2 p45 (NF-E2)-related factor (Nrf2) is a redox-sensitive transcription factor that controls the formation of phase II detoxifying or antioxidant enzymes. This makes Nrf2 activation a key molecular target for many chemopreventive and chemoprotective medications [120]. The bioavailability of EGCG in a specific organ site would determine the degree of DNMT inhibition in vivo. According to the study, EGCG treatment reduced the levels of global DNA methylation in A431 cells in a dose-dependent way. 5-methylcytosine, messenger RNA (mRNA), DNA methyltransferase (DNMT) activity, and the protein levels of DNMT1, DNMT3a, and DNMT3b were all reduced by EGCG. While EGCG reduced methylated H3-Lys 9 levels, it increased acetylated lysine 9 and 14 on histone H3 (H3-Lys 9 and 14) and acetylated lysine 5, 12, and 16 on histone H4. It also decreased histone deacetylase activity. The mRNA and proteins of the suppressed tumor suppressor genes p 16 INK4a and Cip1/p21 were re-expressed as a result of EGCG treatment [121]. While it is anticipated that DNMT inhibition will prevent hypermethylation, recent genetic studies have indicated that severe DNMT activity inhibition may result in DNA hypomethylation, genomic instability, and the early development of cancers like sarcomas and T-cell lymphomas [122,123]. Thus, EGCG has the ability to demethylate tumor suppressor gene promoters, reactivate apoptotic pathways, and change the expression of oncogenic miRNAs by blocking DNMTs [119].

### 4.2. Flavonoids

Flavonoids are polyphenolic compounds widely found in fruits, vegetables, and legumes [124]. It has been shown that flavonoids have a wide variety of anticancer effects. These include regulating the activities of enzymes that scavenge reactive oxygen species, participating in cell cycle arrest, triggering autophagy and apoptosis, and inhibiting the growth and invasion of cancer cells. Flavonoids play two roles in preserving the equilibrium of reactive oxygen species. In healthy physiological settings, they act as antioxidants, but in cancer cells, they also exhibit strong pro-oxidant characteristics. This pro-oxidant activity downregulates pro-inflammatory signaling pathways and triggers apoptotic pathways. The study investigates flavonoids’ biochemical properties, bioavailability, anticancer effectiveness, and modes of action [125]. Flavonoids absorb the hard energy of the ROS and transform it into soft energy by scavenging the radicals. The hard energy that the flavonoids absorb becomes “soft” as a result of passing through the redox modulator. The cell is shielded from a deranged energy flow because this soft energy cannot harm it. The protection sensor, KEAP1, can be effectively activated by the soft energy to produce more antioxidants in the end [126,127].

Quercetin is a member of the flavonoid subclass known as flavonol. It is made up of two benzene rings and an oxygen-containing pyrene ring. Five hydroxyl groups are primarily responsible for its biological activity. It usually takes the form of a glycoside, in which a sugar substituent occupies at least one of those groups. Onions, cabbages, tomatoes, lettuce, radish, pepper, blackcurrant, figs, and many other fruits and vegetables are among the many plant-based foods from which this polyphenol can be extracted [128,129]. When taken orally, quercetin may undergo changes because some of the applied dose is broken down into phenolic acids in the stomach’s acidic environment. The specific glycoside form largely determines how well it is absorbed in the intestines. The bioavailability of quercetin-3-O-oligoglucosides is ten times that of quercetin-3-O-rutinosides, and it is twenty times that of quercetin aglycones [129,130]. In light of this, the majority of approaches to improving quercetin’s effectiveness in the possible treatment of a variety of illnesses, not just cancer, concentrate on changing the structure of quercetin particles or boosting their bioavailability by encapsulating the molecules in various carriers or combining them with other compounds that act as grip points for particular target tissues [131]. Quercetin scavenges ROS at moderate concentrations, preventing oxidative damage to healthy cells [132]. In tumor cells, it can act as a pro-oxidant, increasing ROS beyond a threshold to induce apoptosis. In tumor cells, it may act as a pro-oxidant, raising ROS levels above a certain point to trigger cell death [133]. According to studies, the growth of human leukemia cells was inhibited by the combination of quercetin and hyperoside (QH; 1:1). The findings showed that QH enhanced antioxidant capacity and reduced reactive oxygen species (ROS) production in PC3 cells at a range of concentrations (2.5–60 µg/mL), with peak inhibition and augmentation changes of 3.22 and 3.00 times, respectively. It was discovered that QH increased the expression of tumor suppressor programmed cell death protein 4, which miR-21 antagonistically regulated. The beneficial effect of QH on prostate cancer cells was diminished when pre-miR-21 oligonucleotides were used to induce overexpression of miR-21 [134].

Genistein is an isoflavone derived from soy that has estrogenic, anti-inflammatory, and antioxidant qualities. It shares structural similarities with 17β-estradiol. By altering the estrogen receptor β (ERβ), preventing angiogenesis, stopping the cell cycle, and triggering apoptosis, genistein has anticancer effects. Its antioxidant qualities aid in reducing oxidative stress linked to tumors. Strategies for improving bioavailability, like lipid-based formulations and nanoparticles, exhibit promise. Genistein supports its anticancer effectiveness by influencing epigenetic changes such as DNA methylation and miRNA expression [135]. The PTEN/PI3K/Akt axis is the most prominent oncogenic signaling pathway that genistein modulates at the molecular level. It inhibits downstream survival and proliferative signaling, which stops cancer cells from growing [135]. It is especially pertinent in hormone-dependent diseases due to its capacity to engage with estrogen receptor (ER) signaling. The epigenetic regulation of tumor suppressor genes is one of the most significant ways that genistein prevents breast cancer. Histone modifications at the p21 and p16 promoters and decreased recruitment of the c-MYC–BMI1 complex to the p16 promoter were the mechanisms by which genistein downregulated BMI1 and c-MYC while upregulating p21WAF1 (p21) and p16INK4a (p16). These modifications resulted in the suppression of breast tumorigenesis in xenograft models, the reactivation of tumor suppressor pathways, and selective growth inhibition in precancerous and cancerous breast cells (with minimal effect on normal mammary cells) [136]. It also affects histone acetylation dynamics by modulating histone acetyltransferases (HATs) and histone deacetylases (HDACs), and it inhibits DNA methyltransferases (DNMTs), which reactivates suppressed tumor suppressor genes [137]. Together, these functions support its anti-proliferative and pro-apoptotic properties, inhibit the potential for metastasis, and maintain normal cellular homeostasis in the face of oxidative stress, underscoring its potential as a natural therapeutic agent for the prevention and treatment of cancer.

### 4.3. Alkaloids and Terpenoids

The majority of alkaloids and terpenoids, the largest class of natural products, come from plants. Their anti-tumor effects, which include anti-proliferative, apoptotic, anti-angiogenic, and anti-metastatic activities, are noteworthy among their many biological characteristics. Terpenoids by themselves show how intricately these secondary metabolites trigger autophagy through intricate signaling pathways, including AMPK, NF-kB, PI3K/AKT/mTOR, MAPK/ERK/JNK, and reactive oxygen species. Autophagy induction in tumor cells can have either a protective or destructive effect [138,139]. Epigenetically, berberine reactivates tumor suppressor genes by altering histone acetylation and downregulating oncogenic microRNAs. Together, these effects improve chemosensitivity and inhibit metastasis and proliferation [140]. Sulforaphane, an isothiocyanate found in cruciferous vegetables, has dual redox activity: it activates Nrf2 in normal cells, strengthening antioxidant defenses, while inducing ROS accumulation in cancer cells, triggering apoptosis [141]. Significantly, sulforaphane functions as a natural HDAC inhibitor by influencing the MAPK and PI3K/Akt pathways, encouraging histone acetylation, and reactivating tumor suppressor genes [116]. It regulates microRNA expression and DNA methylation, enhancing its epigenetic control over tumor growth [142]. Alkaloids and terpenoids together demonstrate how plant compounds work as redox-epigenetic modulators. They protect normal cells from oxidative stress while making cancer cells have high levels of ROS and changing their abnormal epigenetic marks.

## 5. Synergistic Role with Conventional Therapies

Antioxidants derived from plants show significant promise as adjuncts in standard cancer treatments [143]. Compounds like resveratrol, quercetin, and curcumin make tumor cells more sensitive to chemotherapy and radiotherapy, which increases the effectiveness of the treatment while reducing the risk of systemic toxicity [144]. For example, resveratrol combined with temozolomide has been shown to improve treatment outcomes in glioblastoma by overcoming resistance mechanisms [145]. Antioxidants also reduce the oxidative stress-induced side effects of chemotherapy drugs, increasing patient tolerance and quality of life [146]. Extensive research has been conducted into the structural basis of MDR activity modulation by flavonoids, as well as the establishment of a strong structure-activity relationship for the ultimate selection of a polyphenolic lead molecule. The P-gp active site was used to test the binding affinities of several synthetic analogs [147]. It was also discovered that several flavonoids can selectively reverse BCRP-mediated drug resistance in various chemoresistant leukemia cell lines. These flavonoid BCRP inhibitors have the potential to improve the efficacy and reduce the toxicity of cancer chemotherapy treatments [148]. Dietary polyphenols were discovered to act as chemopreventive agents by disrupting signal transduction pathways involved in carcinogenesis [149]. Some of the effects of their interference with the cell’s natural processes include cell cycle arrest, apoptosis induction, antioxidant and anti-inflammatory actions, and angiogenesis suppression [150].

## 6. Challenges and Future Perspectives

Polyphenols and other phytochemicals show strong anticancer potential, but their clinical translation remains limited by major challenges. One of the most critical barriers is their low bioavailability, which results from structural modifications during digestion, absorption, and distribution due to interactions with intestinal transporters, digestive enzymes, and plasma proteins. Strategies such as liposomal formulations, nanoparticle encapsulation, and phytochemical conjugates have been proposed to improve tumor-specific accumulation, prolong circulation, and enhance pharmacokinetics. Dosage optimization also remains essential, as many antioxidants exhibit dual redox behavior, acting as pro-oxidants at higher doses and protective agents at lower concentrations. Limited large-scale clinical trials and the heterogeneity of existing studies further hinder clear conclusions regarding therapeutic efficacy.

Despite these challenges, naturally occurring polyphenols—including curcumin, quercetin, EGCG, resveratrol, and apigenin—have demonstrated synergistic effects when combined with chemotherapeutics such as cisplatin, 5-fluorouracil, docetaxel, paclitaxel, and gefitinib. For instance, when compared to free EGCG, EGCG-loaded nanoparticles have been demonstrated to improve intracellular retention, tumor uptake, and plasma half-life. In a similar vein, liposomal curcumin formulations show better stability, longer-lasting release, and increased accumulation in tumor tissues. Polymeric nanoparticles, solid lipid nanoparticles, and nanoemulsions are examples of nanocarrier systems that improve solubility and shield these substances from quick deterioration. These developments demonstrate the potential of nanotechnology-based delivery in overcoming polyphenols’ inherent pharmacokinetic constraints. They modulate multiple hallmarks of cancer by reducing inflammation, inhibiting angiogenesis and metastasis, suppressing proliferation, and inducing apoptosis. Future work should focus on improving clinical applicability through targeted delivery systems and nano-formulations, as well as integrating redox genomics, epigenomics, and metabolomics to guide personalized, biomarker-based interventions. Exploring combinatorial regimens with immunotherapy, gene-editing tools, and epigenetic modulators may further enhance therapeutic outcomes. Advancing tumor-selective, biomarker-driven antioxidant therapies that leverage both redox regulation and epigenetic modulation represents a promising direction for safer and more effective cancer management. Large-scale clinical trials are crucial for validating preclinical findings, standardizing dosing protocols, and identifying patient subgroups that may benefit from antioxidant therapies. Finding predictive biomarkers and comprehending inter-individual variability in response will require integrating multi-omics techniques, such as redox genomics, lipidomics, and single-cell transcriptomics. In order to improve treatment specificity and combat drug resistance, future prospects include combining polyphenols with cutting-edge therapeutic approaches like immune checkpoint inhibitors, CAR-T cells, CRISPR-based gene modulation, and microbiome-targeted interventions.

## 7. Conclusions

Plant-derived antioxidants alter the epigenetic landscape and restore redox balance, making them effective modulators of cancer biology. Their therapeutic significance is highlighted by their capacity to inhibit tumor-suppressive and oncogenic non-coding RNAs, reverse aberrant DNA methylation and histone modifications, and selectively scavenge or induce ROS. These natural compounds, when combined with traditional therapies, not only strengthen anti-tumor effects, but also guard against toxicity related to treatment. In order to verify their clinical value, more mechanistic research, sophisticated delivery methods, and carefully planned clinical trials are required. With further development, antioxidants derived from plants could become a key component of precision oncology, connecting natural medicine with contemporary cancer treatments.

## Figures and Tables

**Table 1:** Clinical studies of plant-derived antioxidants in cancer therapy.

Compounds	Clinical Trial/Study (NCT No. or Reference)	Cancer Type	Intervention and Dose	Key Findings
Curcumin	NCT01246973	Breast Cancer	4 Curcumin C3 Complex 500 mg capsules (2.0 g) taken orally 3 times/day throughout course of radiation treatments plus one week.	curcumin in preventing and/or reducing the severity of dermatitis in radiation treatment site in breast cancer patients.
Curcumin + Piperine	NCT02598726	Reducing Inflammation for Ureteral Stent-Induced Symptoms in Patients with Cancer	Curcumin PO BID or TID; piperine extract PO on days 1–7.	As secondary and exploratory outcomes, the clinical trial examined the best biologically active dose, changes in quality of life, and prostaglandin E2 levels in addition to evaluating the safety and tolerability of curcumin and piperine by identifying adverse events and the maximum tolerated dose.
Resveratrol	NCT00256334	Colon cancer	Patients were randomly assigned to one of four dose cohorts: plant-derived resveratrol tablets (purchased through the Life Extension Foundation, Scottsdale, AZ) at a dose of 80 mg/day, plant-derived resveratrol tablets at a dose of 20 mg/day, Grape Powder (GP) dissolved in water and taken orally (supplied by the California Table Grape Commission) at a dose of 120 g/day, and GP at a dose of 80 g/day.	Test the hypothesis that resveratrol modulates Wnt signaling in vivo in colon cancer and normal colonic mucosa.
EGCG (Green tea extract)	NCT00666562	Nonmetastatic Bladder Cancer	Patients with bladder cancer treated with oral polyphenon E 800 mg EGCG or polyphenon E 1200 mg EGCG once daily for 14–28 days.	The bioavailability of EGCG in bladder tissues, serum, and urine, as well as its impact on catechin levels and associated biomarkers, were assessed in a clinical study; the published summary concentrates on outcome measures rather than particular efficacy result.
EGCG	NCT05039983	Esophageal Cancer	Three times a day, different concentrations of EGCG are dissolved in a 0.9% saline solution. Every time, a fresh batch is created. To ensure that the medication remains in the esophageal walls for an extended period of time, 30 milliliters of the EGCG solution must be swallowed several times.	EGCG has been shown to have antioxidant, anti-inflammatory and anti-tumor effects. The complex effects of EGCG may improve esophageal obstruction during the waiting period before antineoplastic therapy.
Quercetin	NCT01912820	Prostate Cancer	Patients receive GT extract PO BID and quercetin PO BID for 3–6 weeks before undergoing prostatectomy.	Quercetin and Green Tea: A Phase I Randomized, Double-Blind, Placebo-Controlled Two-Arm Study to Increase Green Tea Polyphenol Bioavailability in Men Having Prostate Excision.
Genistein	NCT00244933	Breast Cancer	Novasoy Orally—100 mg 2 times/day for 7 days; 2 times/day on Days 1–21 every 21 days.	For target lesions evaluated by MRI in accordance with the Response Evaluation Criteria In Solid Tumors Criteria (RECIST v1.0): Total Reaction (CR), elimination of every target lesion; Partial Response (PR), a reduction of more than 30% in the total of the target lesions’ longest diameters; CR + PR = Overall Response (OR).
Sulforaphane (Broccoli sprout extract)	NCT00946309	Prostate Cancer	Drug: High Sulforaphane Extract (Broccoli Sprout Extract) 100 umol sulforaphane, every other day for 5 weeks Drug: Microcrystalline Cellulose NF (placebo).	The expression of Phase II detoxification enzymes was marginally impacted by sulforaphane, and hormone-related markers such as DHT, testosterone, and 3α-DG were somewhat decreased. Only minor gastrointestinal adverse events were reported, and it was well tolerated. Oxidative stress and DNA oxidation data were not gathered.

The data from completed clinical trials on sulforaphane, genistein, curcumin, resveratrol, and EGCG that were obtained from ClinicalTrials.gov show how these compounds affect hormone levels, gene expression, safety profiles, and cancer-related biomarkers in human subjects.

## Data Availability

No new data were created or analyzed in this study. Data sharing is not applicable to this article.

## Abbreviations

Akt	Protein Kinase B
BCL2	B-cell lymphoma 2
ceRNA	Competing endogenous RNA
CpG	Cytosine-phosphate-Guanine
DNMT	DNA Methyltransferase
DNMT1	DNA Methyltransferase 1
DNMT3A	DNA Methyltransferase 3A
DNMT3B	DNA Methyltransferase 3B
EMT	Epithelial–Mesenchymal Transition
EGCG	Epigallocatechin Gallate
ERK1/2	Extracellular Signal-Regulated Kinase 1/2
GPX	Glutathione Peroxidase
HAT	Histone Acetyltransferase
HCC	Hepatocellular Carcinoma
HDAC	Histone Deacetylase
HDACs	Histone Deacetylases
HIF-1α	Hypoxia-Inducible Factor 1 Alpha
HMT	Histone Methyltransferase
JNK	c-Jun N-terminal Kinase
lncRNA	Long Non-Coding RNA
MAPK	Mitogen-Activated Protein Kinase
MeCP2	Methyl CpG Binding Protein 2
miRNA	MicroRNA
miR	MicroRNA (prefix for specific miRNAs, e.g., miR-21, miR-34a)
MMP	Matrix Metalloproteinase
mRNA	Messenger RNA
ncRNA	Non-Coding RNA
NETosis	Neutrophil Extracellular Trap Cell Death
NF-κB	Nuclear Factor kappa-light-chain-enhancer of Activated B Cells
NRF2	Nuclear Factor Erythroid 2–Related Factor 2
Nrf2	Nuclear factor (erythroid-derived 2)-like 2 (alternate form)
PI3K	Phosphoinositide 3-Kinase
PDCD4	Programmed Cell Death 4
PTEN	Phosphatase and Tensin Homolog
ROS	Reactive Oxygen Species
SCC	Squamous Cell Carcinoma
SIRT1	Sirtuin 1
SOD	Superoxide Dismutase
SOD2	Superoxide Dismutase 2
SOD3	Superoxide Dismutase 3
TET	Ten-Eleven Translocation (enzymes)
TNFα	Tumor Necrosis Factor Alpha
TSG	Tumor Suppressor Gene
UTR	Untranslated Region
VEGF	Vascular Endothelial Growth Factor
VEGF-A	Vascular Endothelial Growth Factor A
